# A framework for the ethical assessment of chimeric animal research involving human neural tissue

**DOI:** 10.1186/s12910-019-0345-2

**Published:** 2019-01-25

**Authors:** Sebastian Porsdam Mann, Rosa Sun, Göran Hermerén

**Affiliations:** 1 Uehiro Center for Practical Ethics, University of Oxford, Oxford, OX1 1PT UK; 2 Department of Media, Cognition and Communication, University of Copenhagen, DK-2300 Copenhagen S, Denmark; 3 University Hospital of Coventry Clifford Bridge Rd, Coventry, CV2 2DX UK; 4 Department of Medicine, Lund University, Sölvegatan 19, 22100 Lund, Sweden

**Keywords:** Chimeric research, Moral status, Human neural chimeras, Animal research ethics, Cognitive capacities, Dignity, Ethical assessment.

## Abstract

**Background:**

Animal models of human diseases are often used in biomedical research in place of human subjects. However, results obtained by animal models may fail to hold true for humans. One way of addressing this problem is to make animal models more similar to humans by placing human tissue into animal models, rendering them chimeric. Since technical and ethical limitations make neurological disorders difficult to study in humans, chimeric models with human neural tissue could help advance our understanding of neuropathophysiology.

**Main body:**

In this article, we examine whether the introduction of human neural tissue and any consequent cognitive change is relevant to the way we ought to treat chimeras. We argue that changes in cognitive abilities are morally relevant to the extent that they increase the capacities that affect the moral status of any entity, including awareness, autonomy, and sociability. We posit that no being, regardless of species, should be treated in a way that is incommensurate with its moral status. Finally, we propose a framework that can be used to guide ethical assessment of research involving chimeras with advanced cognitive capacities.

**Conclusion:**

We advance this framework as a useful tool for bringing relevant considerations to the forefront for those considering the ethical merit of proposed chimeric research. In doing so, we examine concepts relevant to the question of how any entity may be treated, including moral status, dignity, and capacities.

## Background

Animal models of human diseases are often used in biomedical research in place of human subjects. For example, Parkinson’s disease, a neurodegenerative disorder characterized by progressive loss of motor coordination and associated dopaminergic neurons in the substantia nigra, can be modelled in rats by chemical (6-OHDA) lesions in the rat brain [1]. Novel treatments of Parkinson’s are tested on these models and may progress to trials in human subjects. Much of our understanding of human pathophysiology is derived from animal models. [2–4].

However, results obtained from animal models often fail to hold true for humans. [5] One way of addressing this problem is to make animal models more similar to humans by placing human cells or tissue into animal models, rendering them chimeric. A chimera is “a single organism made up of cells of different embryonic origins” [6]. In 1969 a malignant human tumor was grafted into a mouse, creating the first in vivo animal model of human cancer [7].

Chimeric animals can be powerful tools for research. For example, HIV infection is difficult to model in animals due to immune system differences. Researchers successfully engrafted human lymphoid tissue into mice which developed distinct elements of human immune function [8]. These mice served as better models for AIDS as they supported the development of human T cells - the principal target of the HIV virus. Chimeras are also routinely created to establish pluripotency of stem cells [9].

The research discussed so far is generally thought justifiable if animal welfare and safety protocols are followed. However, certain types of chimeric research are more controversial. In what follows, we use the term ‘chimera’ to refer specifically to one such type of chimera: non-human animals with human neural tissue.

## Main text

Technical and ethical limitations make neurological disorders difficult to study in humans and current animal models are inadequate [10]. Studying human neural tissue in animal models could help address these difficulties, thus advancing our understanding of neuropathophysiology. Chimeras with human neural cells are a closer match in biochemistry and cellular architecture to what is observed in the native human brain. The study of neuronal circuitry, infective viruses or neurodegenerative diseases of humans can be modelled much closer by live human neural cells in chimeras, as opposed to studies of animal brains or cadaveric brains. Undoubtedly, the invasive nature of many investigations would deem them ethically inappropriate to be tested on living humans. In a recent example, researchers demonstrated human-specific pathophysiological features in a chimeric model of Alzheimer’s disease [11].

Given the remarkable integration of human neural tissue in such models, a salient ethical concern is the extent to which human neurons could enhance cognitive capacities in chimeras [12, 13]. This, in addition to the wider ethical implications of chimeric research on society requires careful examination [14]. The ethical frameworks for animal welfare in non-chimeric research have been extensively studied. However, neural-chimeric research poses possibilities and challenges which have not yet been fully analyzed. In particular, chimeras have the potential to acquire human-like or otherwise enhanced cognitive functions; the outcome of chimerization on cognition is largely unpredictable. Such unpredictability and the spectrum of possible outcomes must be taken into account by an ethical framework for the assessment of neural chimeras.

Chimeras with cognitive functions akin to those of a human may sound like science fiction. Indeed, a decade ago Lensch and colleagues thought that “the risk of inadvertently creating a rodent chimera with higher, human brain function is negligible” [9]. But in 2013, Goldman’s lab showed that chimeric mice with human glial cells outperformed litter-mates on measures of learning, memory, and long-term potentiation [15]. Their cognitive abilities were far from human, but the experiment suggests that models with greater cognitive abilities might be possible in the future.

In this article, we examine whether the introduction of human neural tissue and any consequent cognitive change is relevant to the way we ought to treat chimeras. We argue that changes in cognitive abilities are morally relevant to the extent that they increase the capacities that affect the moral status of any entity, including awareness, autonomy, and sociability [16]. We posit that no being, regardless of species, should be treated in a way that is incommensurate with its moral status. Finally, we propose a framework that can be used to guide ethical assessment of research involving chimeras with advanced cognitive capacities. We advance this framework as a useful tool for bringing relevant considerations to the forefront for those considering the ethical merit of proposed chimeric research. In doing so, we examine concepts relevant to the question of how any entity may be treated, including moral status, dignity, and capacities.

### Moral status and moral confusion

In 2003, Robert and Baylis published a study in which they considered whether chimeric research:

“… is objectionable because the existence of such beings would introduce inexorable moral confusion in our existing relationships with nonhuman animals and in our future relationships with [chimeras]” [17].

The potential for confusion referred to concerns the moral status of chimeras. Before we examine the argument put forward by Robert and Baylis, it is worth pausing to consider what they meant. To say that a being has moral status is to say that the being matters morally and has interests which it would be wrong to harm or impede [18–20]. For example, human persons value many facets of experience, such as their continued existence, life projects, relationships, and well-being. On the other extreme, rocks are not sentient, do not have interests, and thus cannot be harmed. Therefore, morally speaking, rocks must be treated differently from rocket scientists; and this is because of their different moral status.

The grounds that give rise to moral status are hotly debated [21]. In their paper, Robert and Baylis distinguished between two types of moral status: animals, in the terminology used in their 2003 article, have moral status *contingent* on the purposes they serve. For example, the moral status of pets is contingent on being their owners’ companions; by contrast, “human beings have an inviolable right to life simply by virtue of being human” - *categorical* moral status [17].

It is important to point out that this is a distinction used and put forward by Robert and Baylis to stimulate thought and discussion. It is not a distinction meant to be taken seriously as a proposed theory of moral status. It is relevant here because of its historical significance and because of the way it illustrates the complicated nature of drawing distinctions between what matters morally and what does not. Given this background, we continue by analyzing without accepting the distinction drawn by Robert and Baylis in the paragraphs below.

The distinction is crucial because contingent moral status does not offer the same protection as.

categorical moral status: contingent moral status is a weaker moral protection, because it relies on the whims and wishes of others, whereas categorical moral status protects human interests from being violated regardless of the wishes of others. According to this theory, the status of non-human animals is not inherent but may be withdrawn when desired or useful. On this view – which we do not endorse - we may slaughter animals, but not humans, for consumption, and the explanation for this is that animals have contingent whereas humans possess categorical moral status.

Chimeras, however, do not fit neatly into either category. To address this issue, it is likely that efforts to assign appropriate moral status to chimeras will be supported and encouraged. The outcomes, however, of such efforts – speculate Robert and Baylis - might also lead to a re-evaluation of the justification for assigning contingent moral status to animals, since the practice of doing so is unlikely to hold up to serious, reasoned scrutiny. Thus, stopping.

chimeric research might be necessary “to protect the privileged place of human animals in the hierarchy of being.” [17].

In fairness to the authors, Robert and Baylis presented these ruminations for discussion, not as an argument that they necessarily agree with themselves. It is not hard to see some fundamental flaws with this view. Firstly, protecting social institutions that depend upon a morally questionable principle is not sufficient reason for continuing to adhere to that principle. What matters is whether the principle is justified, not the inconvenience of changing it. Rather than “introducing confusion into our moral views,” questioning the status of chimeras can “reveal ways in which those views are inadequate and make us think about how we might improve them” [22, 23].

It is worthy of note that the creation of chimeras does not, on the whole, change the species of the animal. Our relationship with chimeras can be analogous to that of the original animal, or a newly discovered species of animal, even with variable cognitive traits. It is also not the case that moral status is simply assigned or taken away, as suggested in Robert and Baylis’ contemplations. A discussion of misconceptions of relevant biological facts in the moral confusion argument is laid out by Haber [24].

### Dignity and moral status

According to the view examined above, humans have categorical moral status simply because they are human. Though we agree that humans possess moral status, the justification given is controversial. Species membership is not necessarily ethically relevant, a point we return to after exploring the related concept of dignity [25]. As argued below, the concepts of moral status and dignity are closely linked.

Dignity is frequently invoked in discussions of part-human chimeras [26, 27]. Whether chimeric research threatens dignity is of great importance as international human rights law prohibits scientific experiments contrary to dignity. If chimeric research, or specific kinds of chimeric research e.g. those involving human neural cells, be judged contrary to dignity under international law, future research into this therapeutically consequential and scientifically important area could not continue legally. This at best implies a severe delay in important advances and at worst implies an abandonment of further investigation or a move away from legitimate, regulated research institutions. In addition, the language and interpretation of EU and UN international human rights law are often heavily normative. Consequently, it is important that these bodies make fully informed decisions based on science and scientists who are aware of and strive to respect the legal and normative obligation to address concerns that may arise concerning respect for dignity.

Dignity is an unfortunately vague concept. For example, it can be held that the sale of body parts, euthanasia or genetic modification should be prohibited because they undermine dignity, but the invocation of dignity without elaboration does not carry any argumentative weight. Macklin once concluded that “appeals to dignity are either vague restatements of other, more precise, notions [such as respect for persons] or mere slogans that add nothing to an understanding of the topic” [28].

However, there are other senses of dignity. In international human rights law, dignity is a foundational concept. The International Bill of Rights (IBR) recognizes: “the inherent dignity and … equal and inalienable rights of all members of the human family [as] the foundation of freedom, justice and peace in the world” [29–31]. Furthermore, the rights contained in these documents “derive from the inherent dignity of the human person” [29–31]. Under this conception, human beings possess intrinsic worth and consequently bear dignity, which in turn grounds human rights. Dignity is shorthand for the moral rights that humans are entitled to because of their inherent worth.

### The grounds of dignity and moral status

If we accept the idea that human interests must be respected because humans possess intrinsic worth, we might ask where this worth comes from. Two possible answers to the question of inherent worth are given by Karpowicz and colleagues in their discussion of dignity and human-to-nonhuman chimeras:

1. “Human dignity [dignity] is a widely shared concept that refers to being worthy or respected because one is human;”

2. “Human dignity [dignity] is based on the recognition that human beings possess, will possess, or have possessed functional and emergent psychological capacities that indicate they are worthy of respect” [32].

What is the difference between these statements? Under 1), being human is sufficient for dignity. Under 2), being human is neither necessary nor sufficient. Under 1), all humans will have dignity, whereas under 2), it is possible that some non-humans may possess dignity and some humans may not.

We argue that 2) is the more satisfactory account. If we were to come across extraterrestrial intelligent life forms, or if advanced capacities of other animals were demonstrated, we would not be justified in harming them simply because they are not human. Rather, their ability to learn and solve complex problems and other traits would demand moral respect.

Under 2), dignity follows from the possession of certain psychological capacities. Although not central to our purposes in this paper, we draw attention to the similarities these capacities have to those which give rise to interests and, thus, moral status. However, to bear dignity, an entity must possess these capacities to a high degree; a degree currently associated only with humans, and, less certainly, higher primates. In contrast to dignity bearers, whom we argue possess enough of the same status-conferring properties underlying full moral status, we argue that beings who possess these capacities to a lesser degree have partial moral status. Partial moral status admits of degrees proportionate to the extent to which the capacities are present and give rise to interests. In the next sections, we sketch an account of dignity and moral status supported by the work of previous theorists.

### Dignity, personhood, moral status and capabilities

Dignity is an important topic not only due to its legal heft but also for further reasons. The first is its similarity to the concept of moral status being conferred by capabilities. The second is the notion that it is the something which grants its possessor a certain worth which must be respected. Thirdly, it reflects a limit on what might otherwise be pure cost-benefit utilitarian calculation: once something possesses dignity, it may no longer be treated as though its wishes are not to be taken into account, whether or not this impedes the greater good. Fourthly, it reflects the kinds of capacities humanity and scholarship have so far identified as being of particular moral importance.

Because of the fundamental importance of these questions, it is important to understand what dignity entails in the present context. We posit that dignity is an attribute attained by the possession of certain capacities – capacities which the vast majority of humans possess and which overlap to a large degree with those thought to confer moral status – which rhetorically, legally and ethically raises the level of an entity to such a degree that their interests must be taken fully into account and that they possess certain rights not available to those without dignity. In the below section, we draw on the work of previous theorists to develop this thesis and attempt to explicate the importance of the consequences of adopting this viewpoint.

Common to the ideas of moral status, personhood and dignity are the ideas that entities are entitled to moral respect because of who they are. But why? Following the previous section, in which we have argued against the view that species membership is a morally relevant determinant of moral worth, we now proceed to our own understanding of the concept and what it might mean for chimeric human neural research.

We posit that moral status stems from a subgroup of a cluster of qualities of experience, understanding, relationality, and other faculties which enable their possessor to pursue activities and lives worthwhile to themselves and others, and/or which enable them to engage with and improve upon the conditions, capabilities and affordances of later lived experiences.

We begin by citing the work of David DeGrazia on personhood, as his writing identifies several of the status-conferring properties argued for in the extant literature. A strong case can be made that full moral status should be accorded to persons. Personhood “is associated with a cluster of more specific properties without being precisely analyzable in terms of any specific subset: autonomy, rationality, self-awareness, linguistic competence, sociability, moral agency, and the capacity for intentional action… A person is someone who has enough of these properties…” [18]. The salient difference is that not all persons may be agents. As DeGrazia explains, we would consider a three-year old child to be a person, though we might not consider them to be an autonomous agent. Some great apes may demonstrate enough psychological capacities to warrant personhood, and others have debated whether chimpanzees can act autonomously [33].

None of these authors suggest we have a clear understanding of where to draw the line between persons and non-persons, and thus between beings with full- and partial moral status. We agree with DeGrazia that we should err side of caution in our treatment of borderline candidates for status, whether they be chimpanzees or chimeras with advanced capacities, so that we do not risk mistreating animals that deserve greater respect [18]. Streiffer explores principles that may be utilized in policy making regarding borderline cases [23], and links the moral status problem of chimeras to moral permissibility of the abortion debate [34].

For the purposes of the argument that follows, we assume without further argument that a person on DeGrazia’s definition has full moral status. DeGrazia mentions several important properties, each of which contributes to the overall determination of personhood. As each of these properties is weakened or removed, so is the case for the personhood of their hypothetical erstwhile possessor. We may infer from this and the general thinking relied upon so far that individuals which do not possess enough of these characteristics are not persons and thus do not have full moral status. For example, an individual – say, a dog - without the capacity for intentional action, much linguistic competence, rationality, and autonomy does not possess personhood and consequently has a lower moral status than an individual who does have these capacities, due to the restricted nature of possible morally significant experiences and actions. In other words, each of the capacities on DeGrazia’s list reflects an important aspect of the capacity to understand, enjoy and contribute in meaningful ways to morally important endeavors.

Thus, such individuals will have only partial moral status. Though dogs, to continue the example, do possess some, even many, of the status-conferring traits mentioned above, they do not possess them to an extent great enough to warrant full moral status or personhood. But since the capacities they do possess are morally relevant, they possess some rather than no moral status.

Dignity under this perception is a special status with both moral and legal aspects. As a matter of law, all humans possess dignity, no matter what capacities they may have. There are strong pragmatic and utilitarian grounds for this. Even in recent history, numerous ideologies and their adherents have justified heinous crimes by reference to the group membership of their victims (i.e. social Darwinism, fascism, national socialism, etc.). A universal human attribution of dignity can perhaps best be understood as a symbol, prophylactic and defense against the possibility of any such abominations in the future.

But as a matter of lifeform ethics, we posit that dignity is a special kind of full moral status; namely one which places a heavy emphasis on the morally relevant capacities of agency, autonomy and rationality. In support of this, we briefly review some contributions to the philosophical theory of dignity current in bioethical and legal discourse.

Kant thought that dignity stems from the capacity to act in accordance with non-instrumental reason, which he termed autonomy, and by which he meant reason not directed at achieving a particular aim (instrumental reason) and freedom of the will.

Working in this tradition, Beyleveld and Brownsword proposed a theory of dignity based on agency, which they define as being able to carry out, or intend to carry out, a voluntary action for a valued outcome [35]. Applying the moral theory of Alan Gewirth, [36] they argue that agents must respect the basic needs of other agents, and it is in this respect that dignity resides. Agents are dignity-bearers because they possess capacities and understanding which grant them interests in life. Consequently, agents must also value the basic conditions required to fulfil their interests – freedom and well-being in the words of Gewirth. It is crucial that agents assist and not interfere with others’ will if they are to fulfill these interests. Dignity arises from this mutual recognition; rights and duties in turn stem from dignity.

More recently, Griffin has advanced a similar account of dignity based on the capacity to form conceptions of a worthwhile life and the ability to pursue such conceptions in practice [37]. Griffin refers to this capacity variously as autonomy, agency or personhood. On his theory, this sort of autonomy is of ultimate importance for all other valued abilities and therefore constitutes the grounds for dignity.

Common to all these theories is the notion that dignity is the property of being worthy of moral respect because of the capacity to formulate and act in accordance with one’s own reason, understanding and deliberation recognition of one’s own interests, and by extension mutual recognition of the interests and dignity of other dignity-bearers.

Dignity-bearers possess certain interests which are made possible only by a self-awareness capable of projecting an identity into the future. Although some entities do not possess the necessary cognitive functions, we imagine that beings capable of experience have an interest in its prolongation. Additionally, the ability to feel pain and distress gives rise to interests in avoiding those sensations. Some animals may have additional interests in the continuation of their social structure. The strength of this interest will vary according to the depth and effect of these sensations and experiences; thus, moral status grounded in interests may also vary according to the possession of cognitive capacities.

Chimeras with advanced capacities would deserve more moral respect than their non-chimeric counterparts, due to their increased understanding and awareness. If chimeras were to possess capacities and interests equal to those of humans, they would possess dignity, and it would be wrong to engage them in research that would be unethical to perform on humans. It is difficult to estimate the likelihood of this occurring. In humans, the higher cognitive capacities necessary for autonomy take decades to develop, and the smaller cranial size of many species may place an upper bound on their potential capacities. The most likely scenario may involve primate chimeras with human neural tissue.

### The principle of commensurability

We draw on the above discussion to posit a minimum requirement for the ethical use of chimeras in research: the principle of commensurability, which states that regardless of how a chimera came into existence or what species it belongs to, the chimera should not be treated in a way that is incommensurate with its moral status. This position has previously been argued by Streiffer in 2005 [16], in the context of advocating for early termination policy for the potential creation of chimeric beings with enhanced moral status.

Because of the need to treat creatures commensurate with their moral status, creating chimeric models with advanced human-type brain functions that ground full moral status would in some ways be self-defeating. The same reasons that prohibit certain kinds of research on humans would also apply to them. Chimeras with enhanced cognition immediately below this level will require stringent justification for use as research subjects.

Possible enhancements of moral status must be assessed in research involving human neural tissue. Many methods have been developed in the comparative and developmental cognition literature for assessing cognition in animals and infants, which may be useful in chimeric cognitive assessments [38–40]. In addition, Aach and colleagues have recently issued a call for more research on the neurobiology underlying capacities relevant to moral status; such studies would provide complementary and highly relevant information for the assessment of commensurability [41, 42]. In the following sections, we sketch a framework for ethical assessment that takes these considerations, as well as other risks, into account.

### Risk and uncertainty

The creation of chimeras holds significant risks for animal welfare due to limited understanding of the resulting biology. For example, chimeric pigs given a specific human growth-associated gene (Beltsville pigs) suffered multiple unforeseen symptoms including diarrhea, lethargy, skin and eye problems and constant arthritic pain [43]. Many animals may suffer before the intended model chimera is created.

A being with greater sentience may also be trapped in its body and suffer greatly if enhanced awareness and understanding is not accompanied by the ability to express them [31]. Detailed discussions regarding the ethics of animal welfare in the context of organ harvesting has been made by Shaw and colleagues [44–47].

Chimeric animals also hold potential risk for humans as they could serve as incubators for diseases. The Ebola virus, influenza and rabies are all examples of zoonoses – diseases transmitted from animals to humans. Zoonoses are a concern for xenograft trials transplanting organs from animal donors into humans as they can be highly virulent.

A range of pathogens have been identified in pig-to-human xenografts, presenting a risk of cross-species infection [48]. Although these risks exist, pre-clinical data suggests that instances will be rare, and as of 2012 no transmissions had occurred [49]. Proposed xenograft trials need to be assessed on their own merits, and steps towards risk minimization should be included. A 2012 World Health Organization consultation recommended a number of preventative steps [﻿50﻿]. Overall, the WHO, the U.S. Food and Drug Administration and several national authorities believe the potential benefits of xenograft research to be large enough to justify careful exploration with safety precautions [49].

Lastly, it is unclear what would happen if chimeric animals were to escape or be released into the wild. Although excellent resources for chemical and biological risk assessment and management exist, [50] much of the necessary information is unavailable due to the novelty and uncertainty involved in chimeric research.

### Precaution and proportionality

The precautionary principle enjoys a long history in several areas of European law. It states that caution should be the preferred approach in cases of scientific advances with wide-spread but unknown effects. The application of the precautionary principle is a matter of debate in the field of ethics. A strict interpretation of the precautionary principle would prohibit the development of chimeras with advanced mental capacities due to their potential risks.

In particular, the principle does not acknowledge proportionality - the possibility that some risk may be justified by countervailing benefits. We suggest the use of an alternative principle - the principle of proportionality, as developed by Hermerén [51]. The principle has, at its core, the notion that means used should not be excessive in relation to the intended goal, and that the option with the greatest balance of benefits over disadvantages should be used. When combined with the principle of commensurability to ensure the moral status and resultant rights of subjects are respected, this principle can be used to sketch recommendations for chimeric research. The principle can be formulated according to four conditions:

(A) Importance of objective - The intended goal should be important for science and/or society.

(B) Relevance of means - The means should bring about or help to achieve the goal.

(C) Most favorable option - There is no less controversial or risky means to achieve the goal.

(D) Non-excessiveness - The means used should not be excessive (resources utilized, opportunity costs, cost to society) in relation to the intended goal.

These conditions capture several important considerations for the responsible advancement of science. (A) underscores the notion that it would be unethical to fund and carry out research unable to bring about an advancement in understanding. The second condition states that proper scientific method must be used to avoid invalidation of experiments by methodological flaws. Condition (C) narrows acceptable research options to those that are less controversial and risky. (C) is a ceteris paribus, not an absolute, clause – that is, other things being equal, the least risky option should be chosen. In situations where a greater benefit can be achieved through a riskier option, condition (C) focuses on the balance of benefit over risk. Finally, condition (D) restrains the choice of means. For example, non-invasive techniques should be chosen over invasive techniques. It would also prohibit the use of unnecessary endpoints, such as protocols for trials that call for very high thresholds of statistical significance.

It should be noted that these criteria will be context-dependent. For example, the standards applicable to applied and to basic science may differ from each other. In basic research, conditions (B) or (C), given the often speculative or fundamental nature of the work, may need to be evaluated with greater leniency compared to applied work. Similarly, the standard for basic research to fulfill conditions (A) and (B) will have to be set in accordance to the scope and likelihood of success of the research in question.

The principle of proportionality is a decision-making tool that incorporates several aspects of good and ethical scientific method in a semi-structured format. It can serve as a useful framework for bringing important issues to the forefront. Each of the conditions highlights an area of concern within chimeric animal research that needs to be addressed by ethical and stem cell review boards.

Chimeric research in general has the potential to bring about important advances in biomedical.

research. However, not all research will satisfy conditions (A) and (B). For example, the creation.

chimeras for the sole purpose of growing tissues to be used in cosmetic as opposed to reconstructive plastic surgery would fail to satisfy condition (A). Conditions (C) and (D) further limit the methodology of chimeric research that are ethically permissible.

Research that can be carried out in vitro, in non-chimeric models or even in humans also prohibit using more sentient chimeras where a less sentient organism will do. These conditions also imply the need for animal protection and risk minimization protocols.

## Conclusion

The creation of non-human chimeras with full moral status is not in itself unethical, but becomes so if the resulting chimeras are treated in a manner incommensurate with their status or would bring about such risks as are likely to outweigh the anticipated benefits [16, 34].

Secondly, the creation of chimeras with partial status for use in research is permissible if they are the best option for answering an important research question. These animals should be treated in accordance with their status, and should not be created for marginally useful discoveries. The use of chimeras with higher, but not full, moral status requires strong justification.

Existing frameworks of animal protection and risk minimization should be extended to.

encompass chimeric animals. Hyun et al. [52, 53] lay out some practical recommendations for considerations in chimeric research, above and beyond frameworks for animal welfare.

Future work should address the repurposing of current capacity assessment tools for chimeric animal research. In the meantime, we have suggested a framework which we hope will be useful in bringing relevant questions to the forefront for those considering the ethical merit of experiments on chimeric animals with human neural tissue.

## Abbreviations

FDA: Food and Drug Administration
IBR: International Bill of Rights
WHO: World Health Organizaiton

## References

[1] Deumens R, Blokland A, Prickaerts J (2002). Modeling Parkinson’s disease in rats: an.evaluation of 6-OHDA lesions of the nigrostriatal pathway. Exp. Neurol..

[2] Houdebine L-M. In target discovery and validation reviews and protocols 163–202: Humana Press; 2007. 10.1385/1%2D59,745%2D165-7:163.

[3] Lieschke GJ, Currie PD (2007). Animal models of human disease: zebrafish swim into view. Nat. Rev. Genet..

[4] Swindle MM, Makin A, Herron AJ, Clubb FJ, Frazier KS (2012). Swine as Models in Biomedical Research and Toxicology Testing. Vet. Pathol..

[5] van der Worp HB (2010). Can animal models of disease reliably inform human studies?. PLoS Med..

[6] Nagy A, Rossant J (2001). Chimaeras and mosaics for dissecting complex mutant phenotypes. Int. J. Dev. Biol..

[7] Rygaard J, Povlsen CO (1969). Heterotransplantation of a human malignant tumour to ‘Nude’ mice. Acta Pathol. Microbiol. Scand..

[8] McCune, J. et al. The SCID-hu mouse: murine model for the analysis of human hematolymphoid differentiation and function. Science (80-). 241, 1632–1639 (1988). 10.1126/science.241.4873.1632 2971269

[9] Lensch MW, Schlaeger TM, Zon LI, Daley GQ (2007). Teratoma formation assays with human embryonic stem cells: a rationale for one type of human-animal chimera. Cell. Stem Cell.

[10] Nestler EJ, Hyman SE (2010). Animal models of neuropsychiatric disorders. Nat. Neurosci..

[11] Espuny-Camacho I (2017). Et al. hallmarks of Alzheimer’s disease in stem-cell-derived human Neurons Transplanted into Mouse Brain. Neuron 93.

[12] Greene M, et al. Moral Issues of Human-Non-Human Primate Neural Grafting. Science (80-.). 2005:309. 10.1126/science.1112207 16020716

[13] Greely HT, Cho MK, Hogle LF, Satz DM (2007). Thinking about the human neuron. mouse. Am. J. Bioeth..

[14] Savulescu J, Beauchamp TL, Frey RG (2013). Genetically Modified Animals: Should There Be Limits to Engineering the Animal Kingdom?. The Oxford Handbook of Animal Ethics (Reprint edition, pp. 641–670). Oxford.

[15] Han X (2013). Forebrain engraftment by human glial progenitor cells enhances synaptic. plasticity and learning in adult mice. Cell Stem Cell.

[16] Streiffer R (2005). At the edge of humanity: human stem cells, chimeras, and moral status. Kennedy Institute of Ethics Journal.

[17] Robert JS, Baylis F (2003). Crossing species boundaries. Am. J. Bioeth.

[18] DeGrazia D (2007). Human-animal chimeras: Human dignity, moral status, and species prejudice. Metaphilosophy.

[19] Buchanan A (2009). Moral status and human enhancement. Philos. Public Aff..

[20] Streiffer R. Human/Non-Human Chimeras. Stanford Encyclopedia of Philosophy. 2014.

[21] Jaworska A, Tannenbaum J. The Grounds of Moral Status. The Stanford Encyclopedia of Philosophy. 2013.

[22] Bok H (2003). What’s wrong with confusion?. Am. J. Bioeth..

[23] Streiffer R (2003). In Defense of the Moral Relevance of Species Boundaries. The American Journal of Bioethics.

[24] Haber MH, Benham B (2012). Reframing the ethical issues in part-human animal research: the unbearable ontology of inexorable moral confusion. The American 172 Journal of Bioethics: AJOB.

[25] Singer, P. Animal Liberation. (Pimlico, 1995).

[26] Palacios-González C (2015). Human dignity and the creation of human-nonhuman chimeras Med. Health Care. Philos..

[27] Eberl JT, Ballard RA (2009). Metaphysical and Ethical Perspectives on Creating Animal-Human Chimeras. Journal of Medicine and Philosophy.

[28] Macklin R (2003). Dignity is a useless concept. BMJ.

[29] United Nations. The Universal Declaration of. Human Rights. 1948.

[30] United Nations (1966). International Covenant on Economic, Social and Cultural Rights.

[31] United Nations (1966). International Covenant on Civil and Political Rights.

[32] Karpowicz P, Cohen CB, van der Kooy D (2005). Developing human-nonhuman chimeras in human stem cell research: ethical issues and boundaries. Kennedy Inst. Ethics J..

[33] Beauchamp TL, Wobber V (2014). Autonomy in chimpanzees. Theor. Med. Bioeth..

[34] Streiffer R (2010). Chimeras, moral status, and public policy: Implications of the abortion debate for public policy on human/nonhuman chimera research. The Journal of Law, Medicine & Ethics.

[35] Beyleveld D, Brownsword R. Human Dignity in Bioethics and Biolaw: Oxford University Press; 2001.

[36] Gewirth A. Reason and Morality: Chicago University Press; 1978.

[37] Griffin J. On Human Rights: Oxford University Press; 2008.

[38] Broom DM (2010). Cognitive ability and awareness in domestic animals and decisions about obligations to animals. Appl. Anim. Behav. Sci..

[39] Parker ST, Mitchell RW, Boccia ML. Self-awareness in animals and humans:Developmental perspectives: Cambridge University Press; 2006.

[40] Gieling ET, Nordquist RE, van der Staay FJ (2011). Assessing learning and memory in pigs. Anim. Cogn..

[41] Aach J, Lunshof J, Iyer E, Church GM. Correction: Addressing the ethical issues raised by synthetic human entities with embryo-like features. Elife. 2017;6. 10.7554/eLife.20674 PMC5360441 28494856

[42] Aach J, Lunshof J, Iyer E, Church GM. Addressing the ethical issues raised by synthetic human entities with embryo-like features. Elife. 2017;6. 10.7554/eLife.20674 PMC5360441 28494856

[43] MacKellar C, Jones DA (2012). Chimera’s Children.

[44] Shaw D (2014). Creating chimeras for organs is legal in Switzerland. Bioethica Forum.

[45] Shaw D, Dondorp W, de Wert G (2014). Using non-human primates to benefit humans: research and organ transplantation. Medicine, Health Care, and Philosophy.

[46] Shaw D, Dondorp W, Geijsen N, de Wert G (2015). Creating human organs in chimaera pigs: an ethical source of immunocompatible organs?. Journal of Medical Ethics.

[47] Palacios-González C. The ethics of killing human/great-ape chimeras for their organs: a reply to Shaw et al. Medicine, Health Care, and Philosophy. 2015. 10.1007/s11019-015-9658-1 PMC4880624 26294174

[48] Mueller NJ, Takeuchi Y, Mattiuzzo G, Scobie L (2011). Microbial safety in xenotransplantation. Curr. Opin. Organ Transplant..

[49] Fishman JA, Scobie L, Takeuchi Y (2012). Xenotransplantation-associated infectious risk: a WHO consultation. Xenotransplantation.

[50] Omenn GS (1997). et al. Framework for Environmental Health Risk Assessment.

[51] Hermerén G (2012). The principle of proportionality revisited: interpretations and applications Med. Health Care. Philos..

[52] Hyun I, Taylor P, Testa G, Dickens B, Jung KW, McNab A (2007). Ethical standards for human-to-animal chimera experiments in stem cell research. Cell Stem Cell.

[53] Hyun I (2013). Bioethics and the Future of Stem Cell Research.

